# Polymorphisms in DNA-Repair Genes in a Cohort of Prostate Cancer Patients from Different Areas in Spain: Heterogeneity between Populations as a Confounding Factor in Association Studies

**DOI:** 10.1371/journal.pone.0069735

**Published:** 2013-07-23

**Authors:** Luis Alberto Henríquez-Hernández, Almudena Valenciano, Palmira Foro-Arnalot, María Jesús Álvarez-Cubero, José Manuel Cozar, José Francisco Suárez-Novo, Manel Castells-Esteve, Adriana Ayala-Gil, Pablo Fernández-Gonzalo, Montse Ferrer, Ferrán Guedea, Gemma Sancho-Pardo, Jordi Craven-Bartle, María José Ortiz-Gordillo, Patricia Cabrera-Roldán, Estefanía Herrera-Ramos, Pedro C. Lara

**Affiliations:** 1 Radiation Oncology Department, Hospital Universitario de Gran Canaria Dr. Negrín, Las Palmas, Spain; 2 Instituto Canario de Investigación del Cáncer, Las Palmas, Spain; 3 Clinical Science Department, Universidad de Las Palmas de Gran Canaria, Las Palmas, Spain; 4 Institud d'Oncologia Radioteràpica, Hospital de la Esperanza, Parc de Salut Mar, Barcelona, Spain; 5 Laboratory of Genetic Identification, Legal Medicine and Toxicology Department, Facultad de Medicina, Universidad de Granada, Granada, Spain; 6 GENYO, Pfizer-University of Granada-Andalusian Government Centre for Genomics and Oncological Research, Granada, Spain; 7 Department of Urology, Hospital Universitario Virgen de las Nieves, Granada, Spain; 8 Department of Urology, Hospital Universitari de Bellvite, L'Hospitalet de Llobregat, Barcelona, Spain; 9 Radiation Oncology Department, Onkologikoa, Guipuzcoa, Spain; 10 Health Services Research Group, Institut de Recerca Hospital del Mar IMIM, Barcelona, Spain; 11 Department of Radiation Oncology, Institut Català d'Oncologia ICO, L'Hospitalet de Llobregat, Barcelona, Spain; 12 Radiation Oncology Department, Hospital de la Santa Creu i Sant Pau, Barcelona, Spain; 13 Radiation Oncology Department, Hospital Universitario Virgen del Rocío, Sevilla, Spain; 14 Immonology Department, Hospital Universitario de Gran Canaria Dr. Negrín, Las Palmas, Spain; Ohio State University Medical Center, United States of America

## Abstract

**Background:**

Differences in the distribution of genotypes between individuals of the same ethnicity are an important confounder factor commonly undervalued in typical association studies conducted in radiogenomics.

**Objective:**

To evaluate the genotypic distribution of SNPs in a wide set of Spanish prostate cancer patients for determine the homogeneity of the population and to disclose potential bias.

**Design, Setting, and Participants:**

A total of 601 prostate cancer patients from Andalusia, Basque Country, Canary and Catalonia were genotyped for 10 SNPs located in 6 different genes associated to DNA repair: XRCC1 (rs25487, rs25489, rs1799782), ERCC2 (rs13181), ERCC1 (rs11615), LIG4 (rs1805388, rs1805386), ATM (rs17503908, rs1800057) and P53 (rs1042522). The SNP genotyping was made in a Biotrove OpenArray® NT Cycler.

**Outcome Measurements and Statistical Analysis:**

Comparisons of genotypic and allelic frequencies among populations, as well as haplotype analyses were determined using the web-based environment SNPator. Principal component analysis was made using the SnpMatrix and XSnpMatrix classes and methods implemented as an R package. Non-supervised hierarchical cluster of SNP was made using MultiExperiment Viewer.

**Results and Limitations:**

We observed that genotype distribution of 4 out 10 SNPs was statistically different among the studied populations, showing the greatest differences between Andalusia and Catalonia. These observations were confirmed in cluster analysis, principal component analysis and in the differential distribution of haplotypes among the populations. Because tumor characteristics have not been taken into account, it is possible that some polymorphisms may influence tumor characteristics in the same way that it may pose a risk factor for other disease characteristics.

**Conclusion:**

Differences in distribution of genotypes within different populations of the same ethnicity could be an important confounding factor responsible for the lack of validation of SNPs associated with radiation-induced toxicity, especially when extensive meta-analysis with subjects from different countries are carried out.

## Introduction

Genetic polymorphisms are variants of the genome that appear by mutations in some individuals, are transmitted to offspring and acquire some frequency (at least 1%) in the population after many generations. Polymorphisms are the basis of evolution and those that are consolidated may be silent or provide benefits to individuals, but can also be involved in disease development [1]. The most frequent polymorphisms are single nucleotide polymorphisms (SNPs). The ethnic origin of a population determines the distribution of genotypes in a population, and has not to be equal to others. Moreover, differences observed within populations of the same ethnic origin suggest that race is not a sufficient factor to ensure the homogeneity of the sample. In that sense, it is known the presence of several significant axes of stratification, most prominently in a northern-south-eastern trend, but also along an east-west axis, among the genotype distribution of European population [2]. In the case of Spain, although populations inhabiting the Iberian Peninsula show a substantial genetic homogeneity [3], there are findings suggesting that Northwest African influences existing among the Spanish populations and these differences might increase the risk for false positives in genetic epidemiology studies [4].

Radiation therapy (RT) is an effective treatment offered to patients with localized prostate cancer as a viable alternative to surgery [5]. Although both therapies showed comparable results in terms of survival [6], the main differences between them are related to adverse effects. Tumour control by RT requires the use of maximum dose that can be delivered while maintaining a tolerance risk of normal tissue toxicity, being clinical toxicity the factor limiting the efficacy of the treatment [7]. The role of genetics in the response of normal tissues to RT is widely accepted by the scientific community, and it would help to explain why patients treated with RT experience a large variation in normal tissue toxicity, even when similar doses and schedules are administered [8]. Radiation causes the loss of structure and function of most biologic molecules, including DNA. The individual DNA repair capacity consists of several mechanisms (nucleotide and base excision repair, homologous recombination, non-homologous endjoining, mismatch repair and telomere metabolism) and the individual capacity to repair damaged DNA may modify the response of tumour tissue and normal tissue to radiation [9]. Thus, studies of candidate genes have been focused on genes mainly involved in DNA damage recognition and repair (eg, ATM, XRCC1, XPD, ERCC1, LIG4, and TP53 among others), and also in free radical scavenging (eg, SOD2), or anti-inflammatory response (eg, TGFB1).

The association between SNPs and radiation toxicity has been deeply explored [10] and numerous consortia have been formed to identify common genetic variations associated with the development of radiation toxicity [11]. Although promising, the overall results failed at the validation stage [12] and today, the development of a SNP signature associated to the prediction of toxicity is still far away. Although this lack of association could be explained by different reasons (presence of confounding factors, insufficient sample size, and lack of consensus in the methodology used in terms of genotyping, statistics, and even in the grading of radiation toxicity) [13], the heterogeneity of the studied populations is a factor whose effect has been commonly underestimated.

With all those assumptions in mind, we designed a study aimed to evaluate the genotypic distribution of 10 SNPs in 6 different genes involved in DNA repair and classically associated to radiation-induced toxicity, in a wide set of Spanish prostate cancer patients, to determine the homogeneity of the population and to disclose potential undervalued confounders in the association between SNPs and radiation toxicity.

## Materials and Methods

### 1. Patients

A total of 601 patients with non-metastatic localized prostate cancer (PCa) were included in the study. Geographical distribution of patients was as follows (Table 1): 91 (15.14%) from Andalusia, 51 (8.48%) from Basque Country, 238 (39.60%) from Canary and 221 (36.77%) from Catalonia. All patients were from Spanish origin and all of them received written informed consent before sample collection. All participants provided their written informed consent to participate in this study. The study was approved by the Research and Ethics Committee of each institution participant in the study: Hospital Universitario de Gran Canaria Dr. Negrín (Las Palmas de Gran Canaria), Hospital de la Esperanza. Parc de Salut Mar (Barcelona), Hospital Universitario Virgen de las Nieves (Granada), Hospital Universitari de Bellvite (L'Hospitalet de Llobregat), Onkologikoa (Guipuzcoa), Institut Català d'Oncologia (L'Hospitalet de Llobregat), Hospital de la Santa Creu i Sant Pau (Barcelona) and Hospital Universitario Virgen del Rocío (Sevilla).

**Table 1:** Regional ancestry of study participants.

Regional ancestry	n	(%)	No. of hospitals
Andalusia	91	(15.14)	2
Basque Country	51	(8.48)	1
Canary	238	(36.60)	1
Catalonia	221	(36.77)	4
Total	601	(100)	8

### 2. DNA Isolation and Quantification

All the blood samples were sent to the Hospital Universitario de Gran Canaria Dr. Negrín for DNA extraction and subsequent analyses. DNA was isolated from 300 µl of whole-blood in an iPrep purification system (Applied Biosystems, Foster City, CA) using the iPrep™ PureLink™ gDNA Blood Kit (Applied Biosystems). DNA was quantified and the quality of samples was determined in a NanoDrop 2000 (Thermo Scientific, Wilmington, DE).

### 3. Genes and SNPs

A total of 10 SNPs in 6 different key genes involved in DNA repair were studied: X-ray repair cross-complementing protein 1 (XRCC1) [14], [15], excision repair cross-complementing rodent repair deficiency, complementation group 2 (ERCC2) [16], excision repair cross-complementing rodent repair deficiency, complementation group 1 (ERCC1) [17], ligase IV (LIG4) [18], ataxia telangiectasia mutated (ATM) [19], and tumour protein p53 (TP53) [20]. Because RT acts producing DNA damage and genetic variation in DNA repair and damage response modify the susceptibility to radiotherapy, these SNPs have been classically associated to radiation-induced toxicity in several tumor types. Description of SNPs is contained in Table 2.

**Table 2:** Description of SNPs included in the study and analyzed by OpenArray.

Gene name	Symbol	Assay ID	SNP ID	Alleles	Chr	Position
**X-ray repair complementing defective repair in Chinese hamster cells 1**						
	XRCC1	C____622564_10	rs25487	C/T	19q13	44055726
	XRCC1	C____622570_10	rs25489	C/T	19q13	44056412
	XRCC1	C__11463404_10	rs1799782	A/G	19q13	44057574
**Excision repair cross-complementing rodent repair deficiency, complementation group 2**						
	ERCC2/XPD	C___3145033_10	rs13181	G/T	19q13	45854919
**Excision repair cross-complementing rodent repair deficiency, complementation group 1**						
	ERCC1	C___2532959_1_	rs11615	A/G	19q13	45923653
**Ligase IV**						
	LIG4	C__11427969_20	rs1805388	A/G	13q33	108863591
	LIG4	C__11427968_10	rs1805386	A/G	13q33	108861913
**Ataxia telangiectasia mutated**						
	ATM	C__33307908_10	rs17503908	G/T	11q22	108215397
	ATM	C__45273750_10	rs1800057	C/G	11q22	108143456
**Tumour protein P53**						
	P53	C___2403545_10	rs1042522	C/G	17p13	7579472

*Abbreviations:* Chr, chromosome; C, cytosine; T, thymine; A, adenine; G, guanine. All the assays were commercially available at Applied Biosystems (see Assay ID).

### 4. Genotyping

The SNP genotyping was made in a Biotrove OpenArray® NT Cycler (Applied Biosystems). DNA for OpenArray (OA) was diluted at a concentration of 50 ng/µl and a ratio of A260/A280 and A260/230 of 1.7–1.9. A total of 300 ng of genomic DNA was used. A final amount of 150 ng was incorporated into the array with the autoloader and genotyped according to the manufacturer's recommendations. A non-template control (NTC) consisting of DNase-free double-distilled water was introduced within each assay. When the DNA and master mix were transferred, the loaded OA plate was filled with an immersion fluid and sealed with glue. The multiplex TaqMan assay reactions were carried out in a Dual Flat Block (384-well) GeneAmp PCR System 9700 (Applied Biosystems) with the following PCR cycle: an initial step at 93°C for 10 minutes followed by 50 cycles of 45 seconds at 95°C, 13 seconds at 94°C and 2 minutes, 14 seconds at 53°C; followed by a final step during 2 minutes at 25°C and holding at 4°C.

**Figure 1:**
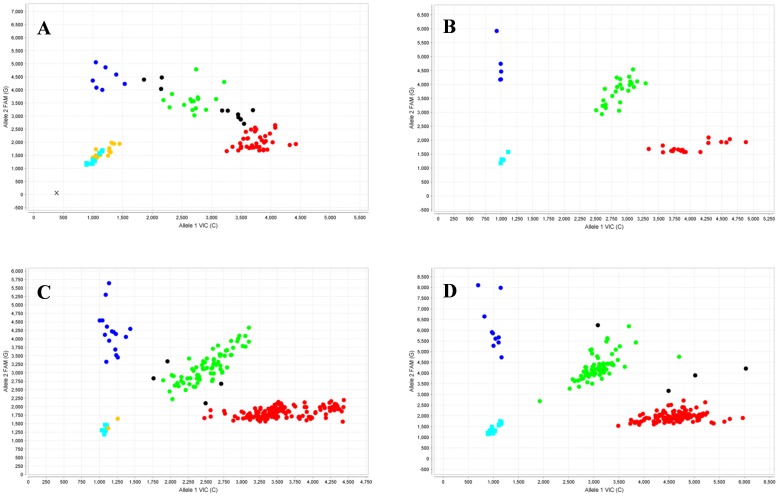
Scatter plots showing genotyping of SNP rs1042522 in (A) Andalusia, (B) Basque Country, (C) Canary, and (D) Catalonia using a Biotrove OpenArray® NT Cycler. Each graph depicts a scattered plot of one allele (FAM probe) versus the other allele (VIC probe). Those samples that are homozygous appear in blue or red; heterozygous samples contain fluorescence from both probes and appear in green. The NTCs appear in light-blue squares and represent the background fluorescence from samples with no template DNA. Samples non-determined appear as black points and samples not amplified appear as orange points. The scatter plots were obtained from the TaqMan Genotyper software version 1.0.1.

The fluorescence results were read using the OpenArray® SNP Genotyping Analysis software version 1.0.5. (Applied Biosystems). The genotyping analysis was made with the TaqMan Genotyper software version 1.0.1. (available at: ttp://www.invitrogen.com/site/us/en/home/Global/forms/taqman-genotyper-software-download-reg.html) using autocalling as the call method. The quality value of the data points genotype was determined by a threshold above 0.95. Genotyping analysis was done for each population separately (Figure 1).

### 5. Statistical Analysis

Genotype and allelic frequencies were determined using the web-based environment SNPator (SNP Analysis To Results, from the Spain's National Genotyping Center and the National Institute for Bioinformatics) [21]. Relative excess heterozygosity was determined to check compatibility of genotype frequencies with Hardy-Weinberg equilibrium (HWE). Thus, p-values from the standard exact HWE lack of fit test were calculated using SNPator. Comparisons of genotypic and allelic frequencies among populations, as well as haplotype analyses were also done in SNPator.

Principal component analysis (PCA) was made using the SnpMatrix and XSnpMatrix classes and methods [22], implemented as an R package and available from Bioconductor (as of version 2.11; http://bioconductor.org). It consists in the transformation of the set of original variables in another set of variables – principal components – obtained as a linear combination of those. The new variables retain all the information, but most of the principal components have so small variability that can be ignored. Thus, few components (generally 3 or less) can represent and explain reasonably the set of objects of the sample without loss of information. PCA reduces the complexity of the data and permits the graphical representation of the variables.

Non-supervised hierarchical clustering [23] of SNP in each population was made using MultiExperiment Viewer (available at: www.tigr.org). Clustering was made using Euclidean distance correlation and average linkage. To success perform the clusters, wild homozygous was encoded as −1, heterozygous as 0 and mutated homozygous as 1.

All additional statistical analyses were performed using PASW Statistics 15 (IBM Corporation, Armonk, NY, USA).

## Results

All the genotyped samples met quality criteria as stated above, and all samples were genotyped with the same batch of material at the same time. A total of 601 PCa patients were genotyped for 10 SNPs. Of the 6,010 possible determinations, 94.36% were successfully genotyped. The call rates among populations were (median (range)): 79.5% (68.1–91.2%) for Andalusia, 100% (80.4–100%) for Basque Country, 97.7% (94.5–99.2%) for Canary, and 97.9% (83.3–99.1%) for Catalonia.

**Figure 2:**
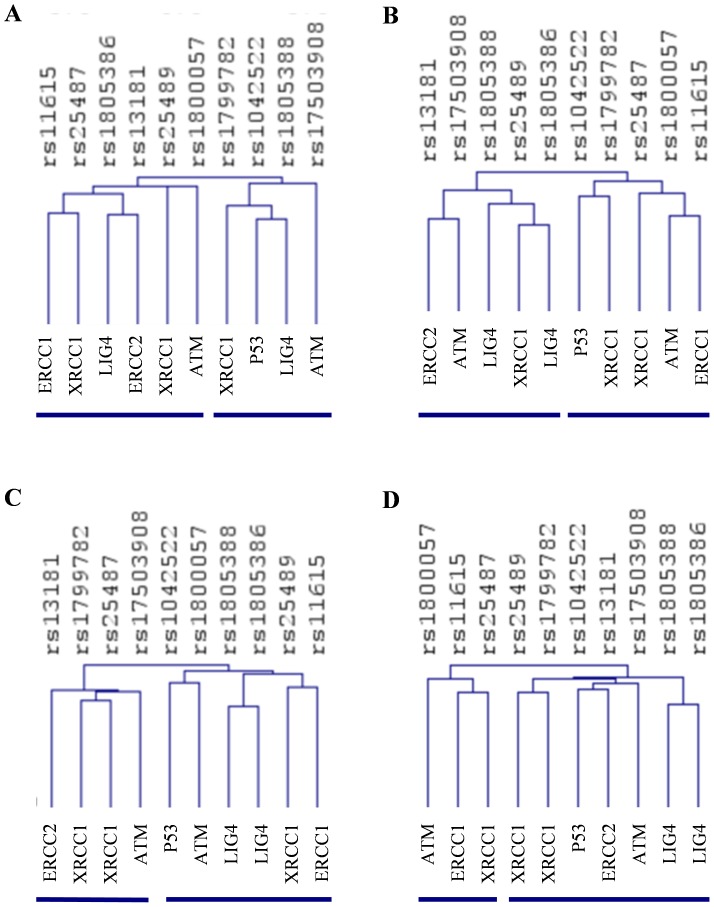
Non-supervised hierarchical clustering of SNPs in prostate cancer patients from (A) Andalusia, (B) Basque Country, (C) Canary and (D) Catalonia. Clustering was made using Euclidean distance correlation and average linkage, and was processed and displayed with MultiExperiment Viewer (http://www.tigr.org). The dendogram shows clustering of SNPs. The gene symbol was added to identify each SNP. Lines below each panel shows the two main clusters generated.

The genotypic and allelic frequencies are shown in Table 3. A relative excess of heterozygosity, indicating a deviation from HWE, was observed in 4 SNPs from 2 different populations: rs25487 (XRCC1) in subjects from Catalonia and rs13181 (ERCC2), rs11615 (ERCC1) and rs180057 (ATM) in subjects from Andalusia (Table 3). The genotype distribution was different between the study populations in 4 of the 10 SNPs: rs25487, rs13181, rs11615, and rs1805386 (LIG4) (χ^2^ test, Table 3), showing a differential distribution of genotypes among populations. A non-supervised hierarchical cluster was performed trying to visualize the differences in the genotype distributions among the four populations. Thus, as shown in Figure 2, polymorphisms were distributed into two main clusters, each one with different number and identity of SNPs, suggesting heterogeneity among populations. Moreover, the web-based tool SNPator was used to compare populations individually one against one. Differences in genotypic distributions were mainly present between Andalusia and the other populations (χ^2^ test, Table 4). According to that result, the populations from Catalonia and Andalusia showed the greatest differences, with 3 SNPs (rs25487, rs13181 and rs11615) differentially distributed among the PCa patients from both populations.

**Table 3:** Genotype and allelic frequencies of gene polymorphisms in this study.

	Call rate	Genotypes			HWE	Alleles	
**XRCC1**							
**rs25487**		**CC**	**CT**	**TT**		**C**	**T**
Andalusia	0.79	0.49	0.34	0.18	ns	0.65	0.35
Basque Country	0.80	0.44	0.51	0.05	ns	0.70	0.30
Canary	0.95	0.48	0.41	0.11	ns	0.68	0.32
Catalonia	0.83	0.36	0.55	0.09	*	0.63	0.37
*P* value				0.012			
**rs25489**		**CC**	**CT**	**TT**		**C**	**T**
Andalusia	0.91	0.81	0.19	0.00	ns	0.90	0.10
Basque Country	1.00	0.86	0.14	0.00	ns	0.93	0.07
Canary	0.97	0.87	0.13	0.00	ns	0.93	0.07
Catalonia	0.98	0.90	0.09	0.01	ns	0.95	0.05
*P* value				0.178			
**rs1799782**		**AA**	**AG**	**GG**		**A**	**G**
Andalusia	0.85	0.00	0.09	0.91	ns	0.05	0.95
Basque Country	1.00	0.00	0.08	0.92	ns	0.04	0.96
Canary	0.99	0.01	0.12	0.87	ns	0.07	0.93
Catalonia	0.98	0.01	0.11	0.88	ns	0.06	0.94
*P* value				0.936			
**ERCC2**							
**rs13181**		**GG**	**GT**	**TT**		**G**	**T**
Andalusia	0.74	0.19	0.15	0.66	*	0.27	0.73
Basque Country	1.00	0.06	0.37	0.57	ns	0.25	0.75
Canary	0.98	0.11	0.45	0.44	ns	0.33	0.67
Catalonia	0.97	0.09	0.53	0.38	ns	0.35	0.65
*P* value				0.0001			
**ERCC1**							
**rs11615**		**AA**	**AG**	**GG**		**A**	**G**
Andalusia	0.70	0.58	0.20	0.22	*	0.68	0.32
Basque Country	1.00	0.43	0.43	0.14	ns	0.65	0.35
Canary	0.98	0.43	0.41	0.16	ns	0.63	0.37
Catalonia	0.99	0.32	0.52	0.16	ns	0.58	0.42
*P* value				0.001			
**LIG4**							
**rs1805388**		**AA**	**AG**	**GG**		**A**	**G**
Andalusia	0.74	0.06	0.12	0.82	ns	0.12	0.88
Basque Country	0.98	0.04	0.38	0.58	ns	0.23	0.77
Canary	0.99	0.03	0.25	0.72	ns	0.15	0.85
Catalonia	0.99	0.05	0.22	0.73	ns	0.16	0.84
*P* value				0.051			
**rs1805386**		**AA**	**AG**	**GG**		**A**	**G**
Andalusia	0.85	0.78	0.16	0.06	ns	0.85	0.15
Basque Country	0.98	0.84	0.16	0.00	ns	0.92	0.08
Canary	0.96	0.73	0.25	0.02	ns	0.85	0.15
Catalonia	0.98	0.66	0.28	0.06	ns	0.80	0.20
*P* value				0.029			
**ATM**							
**rs17503908**		**GG**	**GT**	**TT**		**G**	**T**
Andalusia	0.81	0.03	0.08	0.89	ns	0.07	0.93
Basque Country	0.98	0.00	0.20	0.80	ns	0.10	0.90
Canary	0.99	0.01	0.20	0.79	ns	0.10	0.90
Catalonia	0.98	0.01	0.17	0.82	ns	0.09	0.91
*P* value				0.088			
**rs1800057**		**CC**	**CG**	**GG**		**C**	**G**
Andalusia	0.80	1.00	0.00	0.00	*	1.00	0.00
Basque Country	1.00	0.94	0.06	0.00	ns	0.97	0.03
Canary	0.97	0.95	0.04	0.01	ns	0.97	0.03
Catalonia	0.99	0.92	0.08	0.00	ns	0.96	0.04
*P* value				0.186			
**TP53**							
**rs1042522**		**CC**	**CG**	**GG**		**C**	**G**
Andalusia	0.68	0.63	0.26	0.11	ns	0.76	0.24
Basque Country	1.00	0.41	0.49	0.10	ns	0.66	0.34
Canary	0.97	0.61	0.32	0.07	ns	0.77	0.23
Catalonia	0.98	0.60	0.35	0.05	ns	0.78	0.22
*P* value				0.059			

Statistical differences among genotypes andHardy-Weinberg equilibrium (HWE) are shown. Abbreviations: ns, non-significant. Differences in the genotype distribution were assessed by χ^2^ test. Populations showing no HWE were indicated with an asterisk (*P*<0.01).

**Table 4:** Comparison among populations of allelic and genotypic frequencies.

Comparison	Allelic frequencies	Genotypic frequencies
Can vs. And	–	rs13181
Can vs. Basq	–	–
Can vs. Cat	–	–
And vs. Basq	–	rs1805388
		rs13181
And vs. Cat	–	rs25487
		rs13181
		rs11615
Basq vs. Cat	rs1805386	–

SNPs differentially distributed are shown.

*Abbreviations:* Can, Canary; And, Andalusia; Basq, Basque Country; Cat, Catalonia. The analyses of the genotypic frequencies were performed including the three possible genotypes. Differences were significant with p values <0.01.

**Figure 3:**
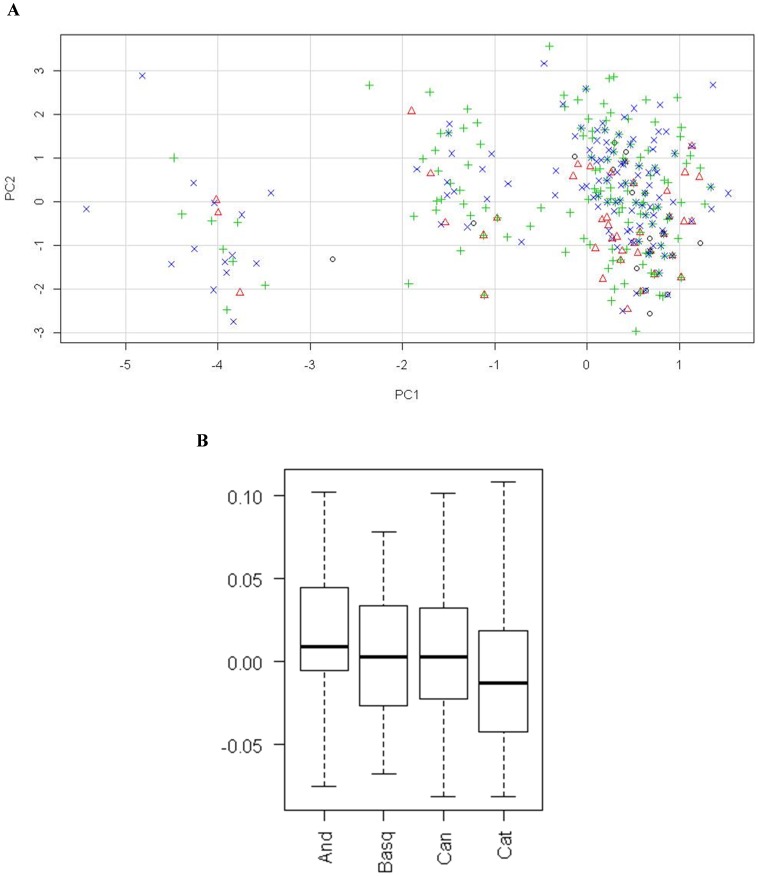
Plot of the top two principal components from the analysis of populations (A), and boxplot of component 1 among the different populations (B). Symbols in plot A: ° (black), Andalusia; Δ (red), Basque Country;+(green), Canary; × (blue), Catalonia. Abbreviations in plot B: And, Andalusia; Basq, Basque Country; Can, Canary; Cat, Catalonia.

Principal component analysis (PCA) was done trying to identify global differences among populations. Components 1 and 2 were responsible for the 15.3% and 14.3% of the variance, respectively. When both components were plotted, the main components seemed not to discriminate between populations (Figure 3A). However, when components were analyzed separately, the first one could distinguish between the populations of Andalusia and Catalonia (Figure 3B), corroborating the results observed in Table 4 and clearly showing the differences in the distribution of genotypes between the analyzed populations.

Finally, haplotype analysis was performed in SNPator. As shown in Table 5, the three most frequent haplotypes were different among populations. Thus, for SNPs in chromosome 11 (those located in ATM gene), the haplotype GG was absent in the Andalusian population. For SNPs in chromosome 13 (those located in LIG4 gene), haplotypes GG and AA showed a different distribution among the populations. In the case of SNPs in chromosome 19 (those located in XRCC1, ERCC2 and ERCC1 genes), haplotype CCGGG was present only in PCa patients from Canary and Catalonia, while haplotype CCGTG was present only in PCa patients from Andalusia and Basque Country. The fact that the most frequent haplotypes were equal in all populations suggests a similarity between individuals of the same ethnicity.

**Table 5:** Analysis of the three most frequent haplotypes (%) in chromosomes 11 (ATM gene), 13 (Lig4 gene) and 19 (XRCC1, ERCC2 and ERCC1 genes) among the studied populations.

Haplotype/chr11	Andalusia	fr	Basque Country	fr	Canary	fr	Catalonia	fr
Hap 1	CT	93.18	CT	89.82	CT	89.15	CT	91.27
Hap 2	CG	6.80	CG	7.24	CG	8.23	CG	4.87
Hap 3	GT	0.02	GG	2.74	GG	2.39	GG	3.86
**Haplotype/chr13**								
Hap 1	AG	76.08	AG	69.81	AG	70.17	AG	64.14
Hap 2	GG	11.89	AA	22.27	AA	15.22	GG	19.60
Hap 3	AA	10.53	GG	7.11	GG	14.49	AA	16.23
**Haplotype/chr19**								
Hap 1	CCGTA	32.52	CCGTA	31.19	CCGTA	27.37	CCGTA	26.11
Hap 2	TCGTA	14.86	CCGTG	14.81	TCGTA	17.03	TCGTA	14.31
Hap 3	CCGTG	11.37	TCGTA	14.29	CCGGG	12.32	CCGGG	12.37

*Abbreviations:* chr, chromosome; fr, frequency; Hap, haplotype. Haplotypes in chr11 is shaped with locus rs1800057 and 17503908, respectively; haplotypes in chr13 is shaped with locus rs1805386 and 1805388, respectively; haplotypes in chr19 is shaped with locus rs25487, rs25489, rs1799782, rs13181, and rs11615, respectively.

## Discussion

Radiogenomics is the study of genetic variants, primarily single nucleotide polymorphisms (SNPs), associated with the development of radiotherapy toxicity, in an attempt to find an assay capable of predicting which cancer patients are most likely to develop adverse effects after RT [9]. The prediction of normal tissue toxicity would allow the adjusting of radiation doses individually for each patient, especially when higher radiation dose levels are associated with improved biochemical control outcomes and reduction in distant metastases in PCa patients [24]. The role of genetics in radiation-toxicity has been proved [25]. In that sense, genetics seem to contribute to explain the high inter-individual variability observed between cases, even when patients are similar and are treated with the same treatment schedule [26]. However, although it has been published a lot of bibliography reporting the predictive role of some SNPs in normal tissue toxicity, the validation studies have failed, calling into question the utility of SNPs as a tool for predicting radiation-induced toxicity [12].

Population association between genotype at a particular locus and a binary trait (such as presence/absence of radiation-induced toxicity) can arise in three ways [27]: i) the locus may be causally related to the disease (different alleles carrying different risks), ii) the locus may not itself be casual (but may be sufficiently close to a causal locus as to be in linkage disequilibrium whit it), or iii) the association may be due to confounding by population stratification or admixture. Confounding may act to create population association in the absence of a casual link or obscure a casual relationship. Thus, it is important to exclude spurious association by appropriate design and/or analysis of studies, taken into account that biases that result from systematic error (such as selection biases or biases in measuring outcomes) persist as the sample size increases. Confounding would arise if the population contained several ethnic groups, if allele frequencies at the locus of interest differed between groups, and if disease frequency also differed between groups for reasons quite unrelated to the locus of interest. It is known that ethnicity influences the applicability of pharmacogenetics [28].

Canary population, as well as the rest of populations included in this study, is considered as Caucasian. However, the natural history of, for example, Canary and Basque Country, are different. Thus, while Canary population has influence from Northwest Africa migration and European colonisation [29], Basques have a different origin [30]. However, in a recent published paper, 30 individuals from 10 different populations from Spain (Canary population was not included in that study) were genotyped for 120 SNPs, concluded that the studied populations were genotypically similar [3]. None of the SNPs considered in the present study were included in this previous article. We found that genotype distribution of 4 SNPs was different among populations from Andalusia, Basque Country, Canary and Catalonia. We compared our findings with the largest cohort of PCa patients analyzed in Spain [31]. A total of 698 Galician PCa patients were screened for 14 SNPs located in the ATM, ERCC2, LIG4, MLH1 and XRCC3 genes. Three of these SNPs were included in our multicenter study: rs1805388 (LIG4), rs1805386 (LIG4) and rs1800057 (ATM). Genotypic distributions of rs1805388 and rs1805386 were significantly different among Galician and the populations included in the present study (χ^2^ test, p = 0.001 and p = 0.007, respectively), highlighting the variability between populations of the same ethnicity (Caucasians) from the same country in depending of each SNP. According to our results, Andalusia was the population differentially distributed, showing the greatest disparity with Catalan (results observed in χ^2^ analyses and PCA). Differences among populations were also evident in haplotype analysis and subsequent distribution. Those results suggest that each SNP need to be considered individually, trying to find possible confounding variables that would be crucial for the interpretation of results. In case-control studies, which is the usual type of design in studies for discovering associations between SNPs and radiation toxicity, the fundamental assumption is that these two series of subjects (controls and cases) may be used to provide unbiased estimates of the corresponding distributions among affected and unaffected members of some underlying population [27]. This fundamental assumption may not be met in practice, leading to biased findings that fall into two broad classes: selection bias caused by inappropriate sampling of cases and controls, and information bias caused by differential measurement errors in cases and controls. When the confounding variable is detected in the study, the classical method in epidemiology is by stratification of the analysis by the potentially confounding variable and testing for association between factors of interest (i.e. genotype) and disease within strata (i.e. grades of radiation-induced toxicity). Concern over the presence of bias from population stratification in genetic case-control studies should be alleviated by proper design and analysis of case-control studies, evaluation of the likelihood of major bias in a given study [32] and, if needed, methods for correction [33].

The present study has some limitations that should be noted. First, all subjects were PCa patients and the genotype frequency may be different if it is compared with a population of healthy subjects. However, in studies designed to evaluate possible associations between SNPs and radiation toxicity, controls are patients with null-low grade of toxicity and cases are patients with high grade of toxicity, but all subjects are cancer patients. Thus, this limitation could be considered as an advantage because it mimics the standard design of such studies. Second, the number of subjects from the different population varies widely. However, the fact that the main differences were not found in the population with the smallest number of patients (Basque Country, with 51 PCa) suggests that this limitation may not be decisive in the interpretation of results. Moreover, if heterogeneity among populations is considered a systematic bias, it is independent of sample size. Third, to blind the analysis, no clinical data on patients were available, that is, there are not data about TNM staging, tumor grade, biochemical failure, or Gleason Score. In that sense, it is possible that some polymorphism may influence tumor characteristics in the same way that it may pose a risk factor for other disease characteristics [34], [35]. In the other hand, some advantages should be highlighted: i) it includes a number of subjects sufficient to have reliable data on the distribution of these 10 SNPs in the PCa populations studied (especially for Canary and Catalonia); ii) all subjects were male, then avoiding the possible bias generated by the gender; and iii) all the determinations (6,010 in total) were performed with the same methodology (OpenArray, Applied Biosystems), with the same batch of chips and by the same investigator, thus minimizing biases from technical origin.

## Conclusions

Differences in distribution of genotypes within different populations of the same ethnicity could be an important confounding factor responsible for the lack of validation of those SNPs associated with radiation-induced toxicity, especially when extensive meta-analysis with subjects from different countries are carried out [36]. Our results suggest that equality between people (especially among those considered as control) should be checked before proceeding with any further analysis.
